# Efficacy and Safety of Immune Checkpoint Inhibitor Combination Therapy for Dysphagia in Patients with Advanced Esophageal Cancer

**DOI:** 10.3390/jcm13164806

**Published:** 2024-08-15

**Authors:** Yurika Nakayama, Takayuki Ando, Hiroaki Takagi, Iori Motoo, Yuko Ueda, Miho Sakumura, Shinya Kajiura, Saeko Takahashi, Seitaro Shimada, Yusuke Takashima, Haruka Fujinami, Kohei Ogawa, Hotaka Tamura, Ayumu Hosokawa, Ichiro Yasuda

**Affiliations:** 1 Third Department of Internal Medicine, University of Toyama, 2630 Sugitani, Toyama 930-0194, Japan; yurika@med.u-toyama.ac.jp (Y.N.); iori4869@med.u-toyama.ac.jp (I.M.); yitaya@med.u-toyama.ac.jp (Y.U.); aomiho@med.u-toyama.ac.jp (M.S.); d12433@med.u-toyama.ac.jp (S.K.); aec56260@hotmail.co.jp (S.T.); sshimada@med.u-toyama.ac.jp (S.S.); tyusuke@shirt.ocn.ne.jp (Y.T.); haruka52@med.u-toyama.ac.jp (H.F.); yasudaic@med.u-toyama.ac.jp (I.Y.); 2 Department of Medical Oncology, Toyama Prefectural Central Hospital, 2-2-78 Nishinagae, Toyama 930-8550, Japan; dxgnj151@yahoo.co.jp (H.T.); kogawa@fuga.ocn.ne.jp (K.O.); 3 Department of Clinical Oncology, University of Miyazaki Hospital, Kihara-5200 Kiyotakecho, Miyazaki 889-1692, Japan; hotaka_tamura@med.miyazaki-u.ac.jp (H.T.); ayhosoka@med.miyazaki-u.ac.jp (A.H.)

**Keywords:** esophageal cancer, dysphagia, immune checkpoint inhibitor, chemotherapy

## Abstract

**Background/Objectives:** Recently, pembrolizumab plus 5-fluorouracil and cisplatin (FP), nivolumab plus FP, and nivolumab plus ipilimumab have become the first-line treatments for patients with advanced esophageal cancer. However, the treatment efficacy in primary tumors has not been reported. We assessed the outcomes of these treatments in advanced esophageal cancer, specifically focusing on esophageal dysphagia improvements and the primary tumor response. **Methods:** This retrospective study was conducted between October 2021 and November 2023. We investigated 23 patients with esophageal cancer and dysphagia who received an immune checkpoint inhibitor (ICI) plus FP or nivolumab plus ipilimumab. **Results:** The median progression-free survival (PFS) was 10.6 months (95% confidence interval [CI]: 9.0–12.5), and the median overall survival was not reached (95%CI: 13.0–NA). Improvement in dysphagia was observed in 19/23 (82.6%) patients, with a median time to improvement of 26 days (range: 15–77 days) and a median dysphagia PFS of 12.6 months (range: 8.1–NA months). Ten patients experienced immune-related adverse events (irAEs): seven had interstitial pneumonia, and three had thyroid dysfunction, pituitary dysfunction, and rash, respectively. **Conclusions:** Although there was a high frequency of irAEs, ICI for esophageal cancer achieved high response rates and prolonged survival. The observed improvement in dysphagia suggests the potential efficacy of the treatment against primary tumors.

## 1. Introduction

Esophageal cancer is the sixth leading cause of cancer-related deaths and the eighth most common type of cancer worldwide [1,2]. Despite advancements in treatment, the prognosis for esophageal cancer remains poor, primarily because most cases are diagnosed at an advanced stage, often accompanied by nutritional disorders and weight loss from inadequate oral intake. Specifically, obstruction due to the primary tumor or extramural pressure from metastatic lymph nodes is a major complication affecting patient quality of life, along with chest pain and bleeding [3].

Recently, pembrolizumab plus 5-fluorouracil (5-FU) and cisplatin (FP), nivolumab plus FP, and nivolumab plus ipilimumab have been approved on the basis of the KEYNOTE-590 [4] and CheckMate 648 trials [5] as the first-line treatments. Currently, an immune checkpoint inhibitor (ICI) plus chemotherapy or nivolumab plus ipilimumab is the standard regimen for unresectable esophageal cancer. However, palliative radiotherapy is recommended for patients with unresectable esophageal cancer presenting with obstruction according to the 2022 esophageal cancer practice guidelines edited by the Japan Esophageal Society [6]. Furthermore, the National Comprehensive Cancer Network guidelines recommend ablation, photodynamic therapy, or stent placement in addition to palliative radiotherapy for dysphagia [3]. This recommendation of radiotherapy is based on several reports demonstrating its efficacy in alleviating symptoms of dysphagia and controlling the primary tumor, although their evidence is weak. Thus, dysphagia, along with performance status (PS), is recognized as an important factor in selecting the first-line treatment. However, in the KEYNOTE-590 [4] and CheckMate 648 trials [5], patients with severe esophageal dysphagia were excluded. Therefore, the efficacy of these treatments, including dysphagia symptoms improvement and the primary tumor response, has not been established.

Makino et al. reported a clinical response rate of 64% in esophageal cancer invading adjacent organs (T4) treated with 5-FU, cisplatin, and docetaxel plus cisplatin and fluorouracil (DCF) induction therapy followed by surgery, compared with 72% for induction chemoradiotherapy (CRT), suggesting that DCF chemotherapy is effective for tumor shrinkage [7]. Moreover, the NExT study showed that the pathological complete response (pCR) rates were 2.2%, 18.6%, and 36.7%, respectively, for locally advanced esophageal cancer treated with FP, DCF, or CRT in neoadjuvant settings [8]. Recently, many clinical trials have investigated the combination of ICI with chemotherapy prior to surgery. Published phase 2 and 3 clinical trials, which included patients with resectable stage I to IV esophageal cancer receiving ICI before surgery, observed a pCR rate of 31.4% and a major pathological response rate of 48.9% [9]. Although these findings were specific to resectable esophageal cancer, they suggest that ICI induces a response in the primary tumor, even in cases of unresectable esophageal cancer.

This retrospective study aims to assess the efficacy and safety of ICI plus chemotherapy or nivolumab plus ipilimumab in patients with advanced esophageal cancer who exhibit dysphagia symptoms. The study specifically focused on improving esophageal dysphagia and primary tumors.

## 2. Materials and Methods

### 2.1. Study Design and Patients

We conducted a multicenter retrospective analysis between October 2021 and November 2023 at three institutions: Toyama University Hospital (Toyama, Toyama, Japan), Toyama Prefectural Central Hospital (Toyama, Toyama, Japan), and the University of Miyazaki Hospital (Miyazaki, Miyazaki, Japan). Esophageal cancer patients with dysphagia who received ICI plus chemotherapy or nivolumab plus ipilimumab regimens were enrolled in this study according to the following eligibility criteria: (1) histologically confirmed esophageal squamous cell carcinoma or adenosquamous carcinoma, (2) unresectable disease with a primary tumor (cStageIVA is also eligible, with or without a target lesion), (3) esophageal dysphagia with a dysphagia score (DS) ≥ 1 (including esophageal dysphagia due to metastatic lymph nodes or the primary tumor), (4) treated with pembrolizumab plus 5-FU and cisplatin, nivolumab plus 5-FU and cisplatin, or nivolumab plus ipilimumab as first-line treatments, (5) no previous ICI treatment, and (6) no previous radiation therapy to the primary lesion.

We reviewed medical records, including sex, age, European Cooperative Oncology Group (ECOG) PS, smoking history, primary site, T stage, histology, disease status (cStageIVA or IVB), PD-L1 status, tumor length, lesion circumference, standard endoscope passage, distant metastasis organ, DS, and nutritional support (intravenous or tube feeding). Tumor-node-metastasis (TNM) staging was classified according to the 8th edition of the Union for International Cancer Control TNM classification and staging system. The ability to swallow was assessed by obtaining the DS, which was defined as follows: 0 for “able to eat normal diet”, 1 for “unable to swallow certain solids”, 2 for “able to swallow semisolid foods”, 3 for “able to swallow liquids only”, and 4 for “unable to swallow liquids” [10].

This study received approval from the institutional review boards of all the participating institutions, including Toyama University Hospital (ethics code: R2023268). The research adhered to the World Medical Association Declaration of Helsinki. An opt-out consent approach, approved by the Review Committee, was used to obtain informed consent from the patients.

### 2.2. Treatments

Patients were treated with pembrolizumab plus 5-FU and cisplatin, or nivolumab plus 5-FU and cisplatin, or nivolumab and ipilimumab. The pembrolizumab plus 5-FU and cisplatin regimen consisted of pembrolizumab 200 mg on day 1 and 5-FU 800 mg/m^2^ on days 1–5 and cisplatin 80 mg/m^2^ on day 1. This regimen was repeated every 3 weeks. The nivolumab plus 5-FU and cisplatin regimen comprised nivolumab 240 mg on day 1 and 5-FU 800 mg/m^2^ on days 1–5 and cisplatin 80 mg/m^2^ on day 1. Nivolumab and chemotherapy were repeated every 2 weeks and 4 weeks, respectively. The nivolumab and ipilimumab regimen included nivolumab 240 mg and ipilimumab 1 mg/kg. Nivolumab and ipilimumab were repeated every 2 weeks and 6 weeks, respectively. The type of regimen was determined by the attending physician on the basis of several factors, including age, PS, tumor volume, and progression rate.

Treatment continued until disease progression, unacceptable toxicity, cancer remission, or the patient’s decision to stop treatment. The 5-FU or cisplatin dosage was adjusted per advanced age, poor PS, grade 4 hematological and grade 3–4 nonhematological adverse events, etc.

### 2.3. Assessments and Statistical Analysis

Tumor response was assessed by performing computed tomography (CT) imaging and in accordance with the Response Evaluation Criteria in Solid Tumors version 1.1. Tumor lesions and lymph nodes were classified as measurable and nonmeasurable based on the following criteria: tumor lesions with a diameter of ≥10 mm and lymph nodes with a short-axis diameter of ≥15 mm were measurable lesions by CT, whereas those not meeting these criteria were nonmeasurable lesions. The following criteria determined response in measurable lesions: complete response (CR) was defined as the total disappearance of all target lesions, with a reduction in measurable and nonmeasurable pathologic lymph nodes to a short-axis diameter of <10 mm. Partial response (PR) was defined as a decrease of at least 30% in the sum of target lesion diameters compared to the baseline measurements. Stable disease (SD) was the lack of adequate shrinkage and sufficient growth to qualify for PR and progressive disease (PD). Progressive disease (PD) was the emergence of new lesions or a minimum of 20% increase in the sum of target lesion diameters compared to the lowest recorded sum since treatment initiation.

For nonmeasurable lesions, including the primary tumor, evaluation with endoscopy was used to define response. CR was defined as the complete disappearance of the primary tumor, confirmed by biopsy. NonCRnonPD was used to define primary tumors without evidence of progression, and PD was used for primary tumors with noticeable progression. In patients with nonCRnonPD, remarkable response (RR) was defined as a remarkable shrinkage in tumor volume to nearly the T1 depth after treatment [11].

The objective response rate (ORR) was defined as the proportion of patients with CR or PR among those with a target lesion. The disease control rate (DCR) was defined as the proportion of patients with CR, PR, or SD. Patients without measurable lesions were excluded from the response rate analysis. Progression-free survival (PFS) was defined as the duration from the first administration of chemotherapy to the radiological or clinical observation of disease progression or death from any cause. Overall survival (OS) was defined as the duration from the first administration of chemotherapy to death from any cause. The maximum tumor shrinkage rate was defined as the maximum change ratio in the tumor diameter after treatment and was evaluated in patients with a target lesion.

The DS was evaluated before treatment and at each patient visit, and we evaluated the passage of an endoscope with a diameter of 8.9–10.4 mm before treatment and at intervals determined by the attending doctor. In JCOG1217, esophageal dysphagia is defined as “the situation with DS ≥ 2 with an inability to pass a standard endoscope (diameter, 9.6–10.4 mm) through the stricture site” [12]. In this study, we defined a standard endoscope as an endoscope with a diameter of 8.9–10.4 mm depending on the type of endoscopy (Olympus Optical Co., Ltd., Tokyo, Japan) used at each institution. The improvement in esophageal dysphagia was assessed by either an improvement in the DS by at least one level or the passage of a standard endoscope (diameter, 8.9–10.4 mm). Additionally, we assessed changes in DS between pre-treatment and the best DS achieved during the first 6 months after treatment initiation. In patients who received salvage radiotherapy within 6 months, only the DS before radiotherapy was evaluated to determine the best DS. Dysphagia PFS was defined as the period between improvement and deterioration of the DS. The withdrawal of nutrition was defined as withdrawal from continuous intravenous infusion or tube feeding for more than a week.

Tumor burden was monitored by performing CT scans every 2 months, with additional scans conducted at the discretion of the attending physician. For cases in which ICIs were discontinued due to immune-related adverse events (irAEs), CT scans were conducted approximately every 3 months. Toxicity and irAEs were classified in accordance with the Common Terminology Criteria for Adverse Events version 5.0. Treatment-related deaths were also assessed. PFS and OS were estimated using the Kaplan–Meier method and compared with the log-rank test. The statistical analysis of the improvement in DS and nutrition status was conducted using a *t*-test. All statistical analyses were performed using EZR version 1.54 (https://www.jichi.ac.jp/saitama-sct/SaitamaHP.files/statmedOSX.html, accessed 23 February 2024).

## 3. Results

### 3.1. Patient Characteristics and Treatment Exposure

A total of 50 patients with unresectable esophageal cancer were treated with either ICI plus chemotherapy or with nivolumab plus ipilimumab. Among them, 27 patients were excluded (9 patients post-chemoradiotherapy, 13 patients post-surgery, and 5 patients with a DS of 0). Therefore, 23 patients with dysphagia received ICI-based treatment. The patients and primary tumor characteristics are shown in Table 1. The median age was 71 (range, 53–89) years. Twenty patients had an ECOG PS of 1, and three had an ECOG PS > 2. Eleven (47.8%) patients were diagnosed with T4 disease. Twenty-one patients were diagnosed with squamous cell carcinoma, while one had adenosquamous carcinoma. Almost all patients were cStageIVB, except for six cStageIVA patients who received ICI plus chemotherapy. Twelve patients had a tumor proportion score (TPS) ≥ 1, and five patients had tumors with a PD-L1 combined positive score (CPS) ≥ 10. CPS or TPS was not evaluated in four patients. Most patients had DSs of 1 or 2, and none were scored as 4. Furthermore, a standard endoscope could not be passed in seven patients. The median tumor length was 5.0 (range, 2–13) cm, with eight patients exhibiting total circumference lesions. Nine patients required nutritional support.

**Figure 1:**
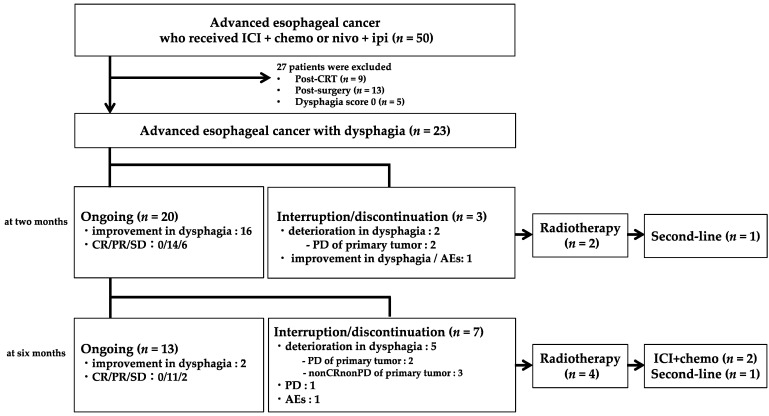
Flow diagram (6 months after treatment). ICI: immune checkpoint inhibitor, chemo: chemotherapy, nivo: nivolumab, ipi: ipilimumab, CRT: chemoradiotherapy, CR: complete response, PR: partial response, SD: stable disease, PD: progressive disease, AEs: adverse events.

Of 23 patients, 22 received ICI plus chemotherapy (16 received pembrolizumab plus FP, and 6 received nivolumab plus FP), and 1 was treated with nivolumab plus ipilimumab. Figure 1 shows the flow diagram 6 months after treatment started. Within 2 months from the start of first-line treatment, it was discontinued in three patients due to PD in the primary tumor (*n* = 2) and adverse events (*n* = 1). Among them, two patients with PD received radiotherapy on the primary tumor. After radiotherapy, one patient was treated with paclitaxel as a second-line treatment. Between 2 and 6 months from the start of first-line treatment, treatment was discontinued in seven patients due to deterioration in dysphagia (two were PD and three were nonCRnonPD in the primary tumor), PD (*n* = 1), and adverse events (*n* = 1). Among them, four patients received radiotherapy. After radiotherapy, two patients resumed first-line treatment, and one was treated with paclitaxel.

In patients receiving ICI plus chemotherapy, the median numbers of treatment cycles for 5-FU, cisplatin, and ICI were 7 (range, 1–19 cycles), 6 (range, 1–9 cycles), and 8 (range, 1–27 cycles), respectively, with a median follow-up period of 11.2 months. Nine patients discontinued treatment due to PD, whereas four patients discontinued treatment due to irAEs, with one patient resuming ICI after irAE treatment. One patient receiving nivolumab plus ipilimumab discontinued treatment after the first course due to irAE.

### 3.2. Efficacy

**Figure 2:**
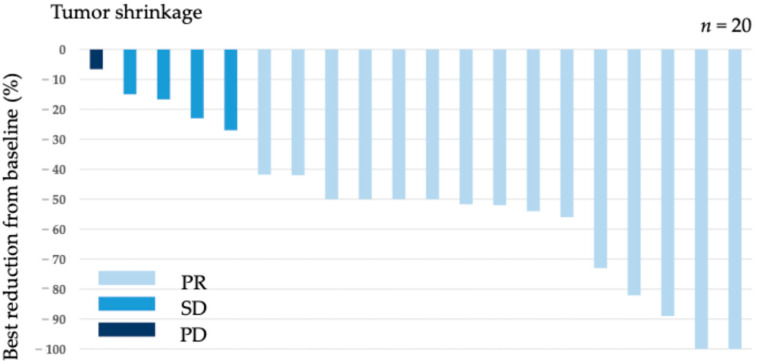
Tumor shrinkage rate for each case.

**Figure 3:**
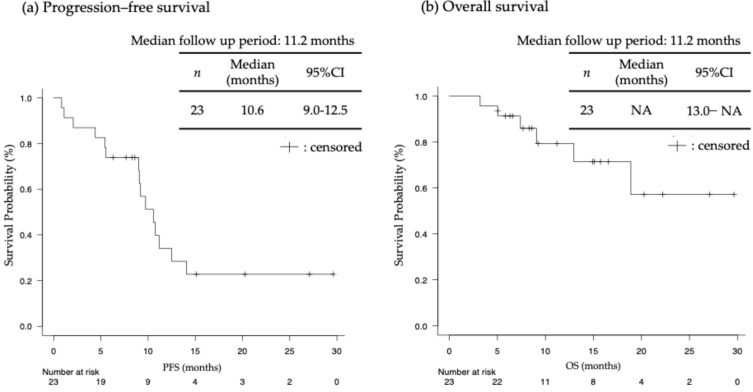
(**a**) Progression–free survival (PFS); (**b**) overall survival (OS) (*n* = 23). NA: not analyzed.

Among 20 patients with a target lesion, 15 achieved PR (ORR = 75.0%). Additionally, four patients achieved SD, leading to a DCR of 95.0% (Table 2). The maximum tumor shrinkage rate in the target lesion for each patient is shown in Figure 2, with a median maximum tumor shrinkage rate of 50% (range, 6.6–100%). In 23 patients with esophageal cancer, the median PFS was 10.6 (95% confidence interval [CI]: 9.0–12.5) and OS was not reached (95%CI: 13.0–NA), with a median follow-up period of 11.2 months (Figure 3).

**Figure 4:**
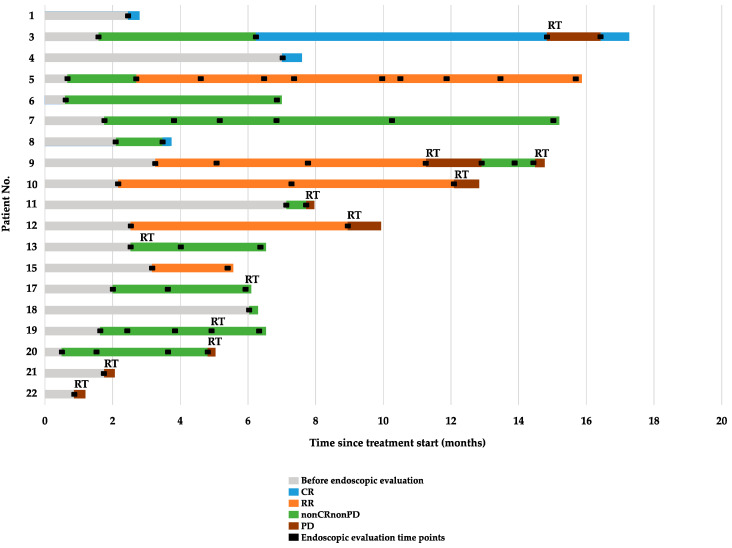
Endoscopic response for each case.

**Figure 5:**
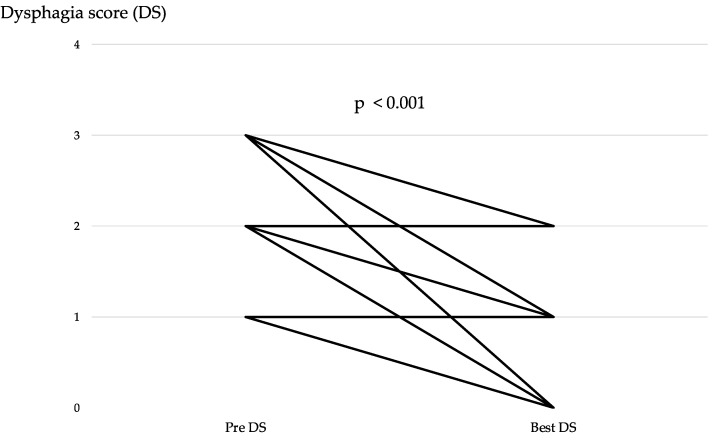
Changes in dysphagia scores during immune checkpoint inhibitor combination therapy.

**Figure 6:**
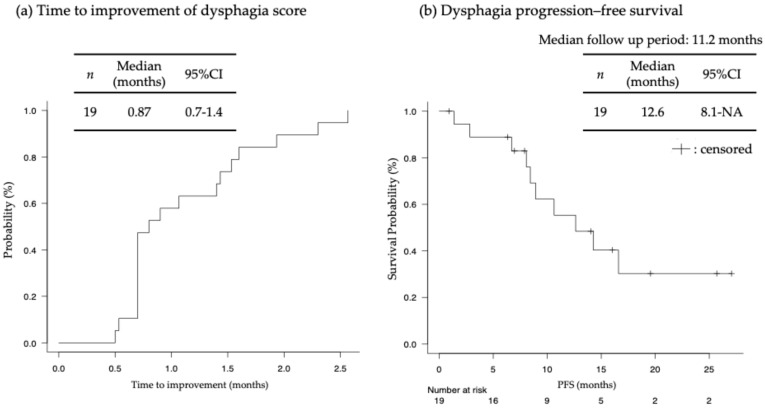
(**a**) Time to improvement of dysphagia score; (**b**) Dysphagia progression–free survival in patients who experienced relief in the dysphagia score by at least one level (*n* = 19). NA: not analyzed. PFS: progression–free survival.

Table 3 and Figure 4 show the response in the primary tumor. CR and RR were observed in four and five patients, respectively. Improvement in the DS by at least one level was observed in 19 (82.6%) of 23 patients, including patients with CR, RR, or nonCRnonPD in the primary tumor. The improvement in the DS between pre-treatment and best response was significant (*p* < 0.001) (Figure 5). Figure S1 shows the changes in DS over the course of treatment. Additionally, a standard endoscope could pass through the esophagus in three (42.8%) out of seven patients, with one patient pending evaluation. Overall, this resulted in an 82.6% dysphagia improvement rate. Furthermore, seven (77.8%) out of nine patients were able to withdraw from nutritional support. Albumin and cholinesterase levels significantly improved post-treatment (*p* < 0.001 and *p* = 0.01, respectively; Figure S2. The median time to improvement in the DS was 26 days (range, 15–77 days), and the median dysphagia PFS was 12.6 months (range, 8.1–NA months) (Figure 6). Dysphagia PFS in patients with CR or RR was probably longer than in those with nonCRnonPD. Among 19 patients who experienced an improvement in DS by at least one level, 7 experienced deterioration in dysphagia due to PD of the primary tumor, but 3 patients’ dysphagia worsened despite having nonCRnonPD. Radiotherapy was added for the primary tumor in seven patients. Additionally, four patients did not experience improvement in dysphagia symptoms after first-line treatment. This lack of improvement was attributed to PD of the primary tumor in three patients and to nonCRnonPD in one patient. Radiotherapy was added for all these patients. As shown in Figure 1 and Figure 4, six patients received salvage radiotherapy within 6 months, and five patients required salvage radiotherapy after 6 months.

Among the patients who received additional radiotherapy, four (36.3%) experienced relief in dysphagia symptoms. However, adverse events were observed in three patients: tracheoesophageal fistula in two (cases 12 and 21) and mediastinitis in one (case 16).

In case 5, the patient had esophageal stenosis due to scarring after ICI combination therapy and required endoscopic dilatation.

### 3.3. Adverse Events

The major grade 3 or 4 adverse events were decreased appetite (21.7%) and neutropenia (17.4%) (Table 4). Additionally, one patient experienced death within 30 days after discontinuing chemotherapy, which occurred because of disease progression following a transfer to best supportive care.

The irAEs were observed in 10 patients: 7 had interstitial pneumonia, and 3 experienced thyroid dysfunction, pituitary dysfunction, and rash, respectively (Table 5). The median time to onset of interstitial pneumonia was 8.16 (range, 2.0–20.6) months. Among seven patients with interstitial pneumonia, first-line treatment was discontinued in all cases. Five patients were treated with prednisolone: two received oral administration and three received intravenous administration. After remediation, ICI was resumed in one patient who experienced grade 1 interstitial pneumonia. Two patients with thyroid dysfunction and pituitary dysfunction were treated with replacement therapy. One patient with a rash was treated with antihistamines. In all patients, the symptoms improved after treatment.

## 4. Discussion

We evaluated the efficacy and safety of ICI plus chemotherapy or nivolumab plus ipilimumab as a first-line treatment for patients with esophageal cancer accompanied by dysphagia. To the best of our knowledge, this is the first study to assess the efficacy of ICI combination therapy in the primary tumor. We observed PFS of 10.6 (95%CI: 9.0–12.5) months, and OS was not reached (95%CI: 13.0–NA). Although these survival periods were longer than those in the KEYNOTE-590 [4] and CheckMate 648 [5] studies, they might be due to the smaller sample size and higher rate of patients with TPS ≥ 1 or CPS ≥ 10 in our study. Furthermore, the improvement rate of dysphagia was 82.6%, with a dysphagia PFS of 12.6 months, and a median time to improvement of 0.87 months.

Dysphagia is an important factor affecting patients’ quality of life and survival, and the DS is a measure of swallowing ability that is typically used to assess esophageal dysphagia. Hagi et al. found that a higher DS (≥3) was considerably associated with a greater incidence of grade 3/4 febrile neutropenia and diarrhea compared to lower scores (≤2). Furthermore, a high DS was associated with a worse clinical response to chemotherapy and a worse 5-year OS [10]. However, as mentioned, the DS is assessed subjectively; therefore, when assessing dysphagia, we also evaluated the passage of a standard endoscope in addition to the DS. Indeed, the stenosis after the endoscopic submucosal dissection of early esophageal cancer is often defined as failure to pass a standard endoscope or subjective symptoms of dysphagia in Japanese clinical trials [12]. In our study, improvement in dysphagia was observed in patients with an endoscopic response, whereas even some patients without an endoscopic response (nonCRnonPD) experienced an improvement in dysphagia. In particular, patients who achieved endoscopic passage after treatment were more likely to have prolonged dysphagia PFS than those without improvement in endoscopic passage. Conversely, some patients with improved dysphagia despite nonCRnonPD required salvage radiotherapy. Therefore, although endoscopic exams may be invasive for patients, an endoscopic response can be a good indicator when considering the follow-up frequency. Careful follow-up is required to determine the timing of salvage radiotherapy, particularly in cases of nonCRnonPD, even when dysphagia has improved.

Several other prospective observational studies and retrospective studies regarding the effect of CRT on the DS have been reported, demonstrating that the improvement rate of the DS ranges from 59% to 91% [13,14,15,16,17,18,19]. Specifically in Japan, it has been reported that the improvement rate of DS by at least one level was 75% with 40 Gy/20 Fr radiation therapy plus cisplatin and 5-FU chemotherapy [19]. In a randomized trial comparing CRT with radiotherapy for unresectable esophageal cancer with dysphagia, the improvement rates of DS were 45% and 38%, respectively. Additionally, in the CRT group, the time to improvement was 9.3 weeks and the dysphagia PFS was 4.1 months, whereas in the radiotherapy group, those times were 9.2 weeks and 3.4 months, respectively [15]. Furthermore, CRT is more likely selected for patients with T4 or severe esophageal dysphagia (DS of 3 or 4). According to several clinical trials conducted mainly in Japan, definitive CRT was established as the effective nonsurgical treatment for unresectable locally advanced esophageal cancer [20,21,22]. Although the CR rate was reported as 62.2% in patients with clinical Stages II–III esophageal cancer who received definitive CRT [23], the rate for unresectable locally advanced esophageal cancer (T4 tumor) was 17–39% [24,25,26,27,28,29]. Therefore, although CRT has been recommended for patients with unresectable esophageal cancer with dysphagia, the choice between ICI combination therapy and CRT in such patients is at the attending physician’s discretion.

In our study, the improvement rates of dysphagia and dysphagia PFS in this study were higher and longer than those previously reported for CRT [15]. Another notable point is that the time to improvement for ICI combination chemotherapy was considerably faster than for CRT. In unresectable esophageal cancer with dysphagia (DS, 1–3), the efficacy of ICI combination therapy in the primary tumor was demonstrated. Additionally, among 23 patients who underwent ICI combination chemotherapy, 11 required salvage radiotherapy. In three of these patients, first-line treatment was resumed after radiotherapy, but the other three patients received second-line treatment with paclitaxel, resulting in a transition rate to chemotherapy after radiotherapy of 54.5%. Thus, approximately half of the patients were able to receive additional radiotherapy following first-line treatment, suggesting that it is possible to prioritize ICI combination chemotherapy even in patients with esophageal dysphagia.

In contrast, the frequency of irAEs was higher in our study than in clinical trials; particularly, interstitial pneumonia was the most frequently diagnosed, with a rate of 30.4%. Aspiration pneumonia and chemical pneumonia, resulting from vomiting or tracheoesophageal fistula, are likely to develop in patients with esophageal cancer. Suazo-Zepeda et al. identified risk factors for irAEs caused by ICI in patients with non-small-cell lung cancer, indicating that smoking is associated with a higher risk of irAEs [30]. Smoking is known to induce changes in the normal immune response patterns and inflammatory processes as well as the recruitment of autoantibodies, leading to a loss of self-tolerance and contributing to the development of irAEs [31,32]. Since almost all patients with esophageal cancer in our study were active or former smokers (82.6%), it is possible that the frequency of interstitial pneumonia was high as well. However, all patients who developed irAEs improved with treatment, and all patients were able to receive chemotherapy or radiotherapy due to adverse events.

In our study, a few cases of worsening dysphagia in some patients were due to scarring associated with tumor shrinkage after treatment. Kim et al. reported predictors of post-treatment stenosis in patients with cervical esophageal cancer undergoing radiotherapy [33]. They found that stage T3/4, total lesion circumference, stenosis at diagnosis, and an endoscopic CR were associated with post-radiotherapy (RT) stenosis in univariate analysis, whereas the total lesion circumference was significant in their multivariate analysis. Dysphagia, as a radiation-induced late esophageal toxicity, is primarily caused by dysmotility and esophageal stricture [34], resulting from muscular damage, submucosal fibrosis, and possibly nerve damage [35]. Reportedly, fibrosis and inflammation of the submucosal and muscular layers are induced by infiltration of inflammatory cells and an increase in local levels of proinflammatory cytokines [36,37,38,39,40], which may explain the significant association between the total lesion circumference and post-RT stenosis. Similar to the previous report, in our study, deterioration of the DS was observed in cases with total circumferential stenosis, possibly due to scarring. However, the association remains uncertain because of our study’s limited sample size.

Furthermore, several studies have demonstrated that OS was significantly shorter in patients with stenosis than in those without stenosis [33,41,42,43,44]. The patients with esophageal stenosis had a poor prognosis because their esophageal stenosis was associated with a higher tumor stage, a larger tumor burden, and a poor nutritional status [43,44,45]. Percutaneous endoscopic gastrostomy (PEG) is a useful nutritional support method for maintaining nutritional status. In our institutions, PEG is typically performed for patients with esophageal dysphagia (DS, 2–4). However, PEG is not performed for patients who have difficulty acquiring tube feeding skills, refuse tube feeding, or lack sufficient familial support for nutritional management with oral supplements and dietary adjustments. Additionally, total parenteral nutrition is introduced instead of PEG for patients requiring immediate treatment initiation, such as those with high tumor volumes or respiratory symptoms due to tracheal invasion.

Therefore, adverse events caused by ICIs are often manageable, allowing for the initiation of ICI combination chemotherapy with nutritional management. However, this study did not include any patients with a DS of 4, suggesting that radiotherapy should be recommended in such cases, but further investigation is needed regarding the degree of stenosis.

Some limitations of this study need to be acknowledged. First, this was a retrospective study, which made it challenging to ensure the accuracy of the collected data regarding the DSs. Second, the follow-up period for OS might have been insufficient to capture long-term outcomes adequately. Third, the objective assessment of the primary tumors was based on endoscopic evaluation. Other objective methods, such as volumetric measurement of lesions using CT images, could be considered. Fourth, although we compared nutritional status between the pre- and post-treatment phases, quality-of-life measures were not evaluated. Additionally, the sample size was small. Therefore, a prospective study has been initiated, and further analyses of the long-term efficacy and safety will be considered as more cases are accumulated in the future.

## 5. Conclusions

Although ICI has demonstrated high response rates and prolonged survival in advanced esophageal cancer, it is important to note the high frequency of irAEs. Improvement in esophageal dysphagia has been observed, suggesting the potential effectiveness of ICI for the primary tumor as well.

## Figures and Tables

**Table 1:** Patient and primary tumor characteristics.

			*n* = 23 (%)
sex		male/female	16/7 (69.6/30.4)
age (years)		median (range)	71 (53–89)
performance status (ECOG)		0/1/2/3	0/20/1/2 (0/86.9/4.3/8.7)
smoking history		−/+	4/19 (17.4/82.6)
primary site		Ce/Ut/Mt/Lt/EGJ	0/6/11/5/1 (0/26.1/47.8/21.7/4.3)
T stage		1/2/3/4	0/2/10/11 (0/8.7/43.5/47.8)
histologic type		squamous cell carcinoma/others	22/1 (95.7/4.3)
disease status		CstageIVa/IVb	6/17 (26.1/73.9)
PD-L1 status	TPS (13/23)	<1/≥1	1/12 (7.7/92.3)
	CPS (6/23)	<10/≥10	1/5 (16.7/83.3)
tumor length (cm)		median (range)	5 (2–13)
circumference of the lesion		<1/2/1/2–3/4/total	3/12/8 (13/52.2/34.8)
passage of a standard endoscope		success/failure	16/7 (69.6/30.4)
metastatic sites		lymph node/lung/liver	16/6/3 (69.6/26.1/13)
dysphagia score		1/2/3/4	8/12/3/0 (34.8/52.2/13/0)
nutritional support (9/23)		intravenous nutrition/tube feeding	4/5 (44.4/55.6)

Ce: cervical esophagus, Ut: upper thoracic esophagus, Mt: middle thoracic esophagus, Lt: lower thoracic esophagus, EGJ: esophagogastric junction, TPS: tumor proportion score, CPS: combined positive score, ECOG: European Cooperative Oncology Group.

**Table 2:** Response for the target lesion (*n* = 20).

	CR	PR	SD	PD	Response Rate (%)	Disease Control Rate (%)
response for the target lesion	0	15	4	1	75.0% (15/20)	95.0% (19/20)

CR: complete response, PR: partial response, SD: stable disease, PD: progressive disease.

**Table 3:** Primary tumor outcomes in each case (*n* = 23).

Case	Age/Sex	T	Circ	Response (RECIST)	Response (Endoscopy)	Endoscopic Passage		Dysphagia Score		Dysphagia PFS (m)	Salvage RT (Gy)	PFS (m)	OS (m)
						**Pre**	**Post**	**Pre**	**Best**				
1	64/M	4	1/3	PR	CR	S	S	3	0	27.1 *	-	29.6 *	29.6 *
2	66/M	4	total	PR	NE	F	NE	3	1	25.7 *	-	27.1 *	27.1 *
3	53/F	2	1/3	PR	CR	S	S	1	0	12.6 (PD)	40 **	14.1	22.2 *
4	71/M	4	1/3	PR	CR	S	S	2	0	19.6 *	-	20.3 *	20.3 *
5	63/F	4	total	PR	RR	F	F	3	2	16.6 (nonCRnonPD)	-	12.5	18.9
6	73/M	4	1/2	SD	nonCRnonPD	S	S	2	0	16.0 *	-	9.7	16.5 *
7	73/M	3	2/3	PR	nonCRnonPD	S	S	2	1	14.3 (PD)	-	10.8	15.7 *
8	72/F	4	1/2	PR	CR	F	S	2	0	14.0 *	-	15.1 *	15.1 *
9	77/M	3	1/2	nonCRnonPD	RR	S	S	1	0	8.9 (PD)	20 **	10.6	14.9 *
10	89/F	3	1/2	PR	RR	F	S	2	1	10.6 (PD)	50.4 **	11.2	13.0
11	74/M	3	1/2	PR	nonCRnonPD	S	NE	2	0	8.1 (PD)	60 **	9.2	11.2 *
12	68/M	3	2/3	PR	RR	S	S	1	0	8.4 (PD)	30 **	9.0	9.2 *
13	82/M	4	1/2	PR	nonCRnonPD	F	F	2	2	−(nonCRnonPD)	50.4	9.1	9.1
14	70/M	3	total	PR	NE	S	NE	1	0	7.9 *	-	8.6 *	8.6 *
15	74/F	3	total	SD	RR	S	S	1	0	6.3 *	-	8.3 *	8.3 *
16	70/F	4	total	PR	NE	S	NE	1	0	7.0 *	-	7.7 *	7.7 *
17	67/M	4	total	SD	nonCRnonPD	F	S	2	1	6.7 (nonCRnonPD)	41.4	5.4	7.4
18	67/M	3	1/2	PR	nonCRnonPD	S	NE	1	0	0.9 *	-	5.5	6.6 *
19	75/M	4	3/4	SD	nonCRnonPD	S	S	2	1	3.1 (nonCRnonPD)	60	6.3 *	6.3 *
20	71/M	2	total	nonCRnonPD	nonCRnonPD	S	F	2	2	−(PD)	50	4.4	5.8 *
21	68/F	3	1/2	PD	PD	S	F	1	1	−(PD)	50	1.1	5.0
22	59/M	4	total	nonCRnonPD	PD	F	F	2	2	−(PD)	30	0.8	5.0 *
23	71/M	3	1/2	PR	NE	S	NE	2	1	1.4 (PD)	-	2.1	3.2

T: T stage, Circ: circumference of the lesion, RR: remarkable response, Passage: passage of a standard endoscope (at diagnosis → after treatment), S: success, F: failure, NE: not evaluated, pre: pre–treatment, post: post–treatment, best: best dysphagia score after first-line treatment, dysphagia PFS: dysphagia progression-free survival (months), RT: radiotherapy, ** salvage radiotherapy performed more than 6 months after initiation of combination therapy with an immune checkpoint inhibitor, PFS: progression–free survival (months), OS: overall survival, * censored data.

**Table 4:** Adverse events (*n* = 23).

Grade	Any (%)	1–2	3	4
leukopenia	13 (56.5)	13	0	0
neutropenia	14 (60.1)	10	3	1
febrile neutropenia	0 (0)	0	0	0
anemia	18 (78.3)	18	0	0
thrombocytopenia	7 (30.4)	7	0	0
nausea	13 (56.5)	13	0	0
vomiting	3 (13.0)	3	0	0
decreased appetite	21 (91.3)	16	5	0
fatigue	11 (47.8)	11	0	0
stomatitis	9 (39.1)	9	0	0
diarrhea	3 (13.0)	3	0	0
constipation	7 (30.4)	7	0	0
peripheral neuropathy	2 (8.7)	2	0	0

**Table 5:** Immune-related adverse events (*n* = 23).

Grade	Any (%)	1–2	3	4
interstitial pneumonia	7 (30.4)	5	1	1
thyroid dysfunction	1 (4.3)	1	0	0
pituitary dysfunction	1 (4.3)	1	0	0
rash	1 (4.3)	1	0	0

## Data Availability

All data are included in the article.

## Supplementary Materials

The following supporting information can be downloaded at: https://www.mdpi.com/article/10.3390/jcm13164806/s1, Figure S1: The change in dysphagia scores for each case. Figure S2: The change in nutrition status.

## References

[1] Lagergren J., Smyth E., Cunningham D., Lagergren P. (2017). Oesophageal cancer. Lancet.

[2] Ouyang G., Liu Q., Wu Y., Liu Z., Lu W., Li S., Pan G., Chen X. (2017). The global, regional, and national burden of gallbladder and biliary tract cancer and its attributable risk factors in 195 countries and territories, 1990 to 2017: A systematic analysis for the global burden of disease study 2017. Cancer.

[3] (2024). NCCN Guidelines for Patients Esophageal Cancer, Version 2. https://www.nccn.org/patientresources/patient-resources/guidelines-for-patients.

[4] Sun J.M., Shen L., Shah M.A., Enzinger P., Adenis A., Doi T., Kojima T., Metges J.P., Li Z., Kim S.B. (2021). Pembrolizumab plus chemotherapy versus chemotherapy alone for first-line treatment of advanced oesophageal cancer (KEYNOTE-590): A randomised, placebo-controlled, phase 3 study. Lancet.

[5] Doki Y., Ajani J.A., Kato K., Xu J., Wyrwicz L., Motoyama S., Ogata T., Kawakami H., Hsu C.-H., Adenis A. (2022). Nivolumab combination therapy in advanced esophageal squamous-cell carcinoma. N. Engl. J. Med..

[6] Kitagawa Y., Ishihara R., Ishikawa H., Ito Y., Oyama T., Oyama T., Kato K., Kato H., Kawakubo H., Kawachi H. (2023). Esophageal cancer practice guidelines 2022 edited by the Japan esophageal society: Part 1. Esophagus.

[7] Makino T., Yamasaki M., Miyazaki Y., Wada N., Takahashi T., Kurokawa Y., Nakajima K., Takiguchi S., Mori M., Doki Y. (2018). Utility of initial induction chemotherapy with 5-fluorouracil, cisplatin, and docetaxel (DCF) for T4 esophageal cancer: A propensity score-matched analysis. Dis. Esophagus.

[8] Kato K., Machida R., Ito Y., Daiko H., Ozawa S., Ogata T., Hara H., Kojima T., Abe T., Bamba T. (2023). A randomized controlled phase III trial comparing doublet chemotherapy, triplet chemotherapy, and doublet chemotherapy combined with radiotherapy as neoadjuvant treatment for locally advanced esophageal cancer: The JCOG1109 next study. Lancet.

[9] Ge F., Huo Z., Cai X., Hu Q., Chen W., Lin G., Zhong R., You Z., Wang R., Lu Y. (2022). Evaluation of clinical and safety outcomes of neoadjuvant immunotherapy combined with chemotherapy for patients with resectable esophageal cancer. JAMA Netw. Open.

[10] Hagi T., Makino T., Yamasaki M., Tanaka K., Nishida N., Sakai D., Motoori M., Kimura Y., Satoh T., Mori M. (2019). Dysphagia score as a predictor of adverse events due to triplet chemotherapy and oncological outcomes in 434 consecutive patients with esophageal cancer. Ann. Surg. Oncol..

[11] Mine S., Tanaka K., Kawachi H., Shirakawa Y., Kitagawa Y., Toh Y., Yasuda T., Watanabe M., Kamei T., Oyama T. (2024). Japanese Classification of Esophageal Cancer 12th edition. Jpn. Esophageal Soc..

[12] Mizutani T., Tanaka M., Eba J., Mizusawa J., Fukuda H., Hanaoka N., Takeuchi M., Aoyama I., Kojima T., Takizawa K. (2015). A phase III study of oral steroid administration versus local steroid injection therapy for the prevention of esophageal stricture after endoscopic submucosal dissection (JCOG1217, Steroid EESD P3). Jpn. J. Clin. Oncol..

[13] Coia L.R., Soffen E.M., Schultheiss T.E., Martin E.E., Hanks G.E. (1993). Swallowing function in patients with esophageal cancer treated with concurrent radiation and chemotherapy. Cancer.

[14] Urba S.G., Turrisi A.T. (1995). Split-course accelerated radiation therapy combined with carboplatin and 5-fluorouracil for palliation of metastatic or unresectable carcinoma of the esophagus. Cancer.

[15] Hayter C.R.R., Huff-Winters C., Paszat L., Youssef Y.M., Shelley W.E., Schulze K. (2000). A Prospective trial of short-course radiotherapy plus chemotherapy for palliation of dysphagia from advanced esophageal cancer. Radiother. Oncol..

[16] Harvey J.A., Bessell J.R., Beller E., Thomas J., Gotley D.C., Burmeister B.H., Walpole E.T., Thomson D.B., Martin I., Doyle L. (2004). Chemoradiation therapy is effective for the palliative treatment of malignant dysphagia. Dis. Esophagus.

[17] Burmeister B.H., Walpole E.T., Burmeister E.A., Thomas J., Thomson D.B., Harvey J.A., Smithers B.M., Gotley D.C. (2005). Feasibility of chemoradiation therapy with protracted infusion of5-fluorouracil for esophageal cancer patients not suitable for cisplatin. Int. J. Clin. Oncol..

[18] Cho S.H., Shim H.J., Lee S.R., Ahn J.S., Yang D.H., Kim Y.K., Nam T.K., Lee J.J., Kim H.J., Chung I.J. (2008). Concurrent chemoradiotherapy with S-1 and cisplatin in advanced esophageal cancer. Dis. Esophagus.

[19] Ikeda E., Kojima T., Kaneko K., Minashi K., Onozawa M., Nihei K., Fuse N., Yano T., Yoshino T., Tahara M. (2011). Efficacy of concurrent chemoradiotherapy as a palliative treatment in Stage IVB esophageal cancer patients with dysphagia. Jpn. J. Clin. Oncol..

[20] Ishida K., Ando N., Yamamoto S. (2004). Phase II study of cisplatin and 5-fluorouracil with concurrent radiotherapy in advanced squamous cell carcinoma of the esophagus: A Japan esophageal oncology group (JEOG)/Japan clinical oncology group trial (JCOG9516). Jpn. J. Clin. Oncol..

[21] Ishikura S., Ohtsu A., Shirao K., Muro K., Kagami Y., Nihei K., Mera K., Ito Y., Boku N., Yoshida S. (2005). A phase I/II study of nedaplatin and 5-fluorouracil with concurrent radiotherapy in patients with T4 esophageal cancer: Japan clinical oncology group trial (JCOG 9908). Esophagus.

[22] Shinoda M., Ando N., Kato K., Ishikura S., Kato H., Tsubosa Y., Minashi K., Okabe H., Kimura Y., Kawano T. (2015). Randomized study of low-dose *versus* standard-dose chemoradiotherapy for unresectable esophageal squamous cell carcinoma (JCOG0303). Cancer Sci..

[23] Kato K., Muro K., Minashi K., Ohtsu A., Ishikura S., Boku N., Takiuchi H., Komatsu Y., Miyata Y., Fukuda H. (2010). Phase II Study of chemoradiotherapy with 5-fluorouracil and cisplatin for stage II–III esophageal squamous cell carcinoma: JCOG trial (JCOG 9906). Int. J. Radiat. Oncol. Biol. Phys..

[24] Seto Y., Chin K., Gomi K., Kozuka T., Fukuda T., Yamada K., Matsubara T., Tokunaga M., Kato Y., Yafune A. (2007). Treatment of thoracic esophageal carcinoma invading adjacent structures. Cancer Sci..

[25] Fujita H., Sueyoshi S., Tanaka T., Tanaka Y., Matono S., Mori N., Shirouzu K., Yamana H., Suzuki G., Hayabuchi N. (2005). Esophagectomy: Is it necessary after chemoradiotherapy for a locally advanced t4 esophageal cancer? Prospective nonrandomized trial comparing chemoradiotherapy with surgery versus without surgery. World J. Surg..

[26] Kaneko K., Ito H., Konishi K., Kurahashi T., Ito T., Katagiri A., Yamamoto T., Kitahara T., Mizutani Y., Ohtsu A. (2003). Definitive chemoradiotherapy for patients with malignant stricture due to T3 or T4 squamous cell carcinoma of the oesophagus. Br. J. Cancer.

[27] Nishimura Y., Suzuki M., Nakamatsu K., Kanamori S., Yagyu Y., Shigeoka H. (2002). Prospective trial of concurrent chemoradiotherapy with protracted infusion of 5-fluorouracil and cisplatin for T4 esophageal cancer with or without fistula. Int. J. Radiat. Oncol. Biol. Phys..

[28] Itoh Y., Fuwa N., Matsumoto A., Asano A., Morita K. (2001). Outcomes of radiotherapy for inoperable locally advanced (T4) esophageal cancer-retrospective analysis. Radiat. Med..

[29] Ohtsu A., Boku N., Muro K., Chin K., Muto M., Yoshida S., Satake M., Ishikura S., Ogino T., Miyata Y. (1999). Definitive chemoradiotherapy for T4 and/or M1 lymph node squamous cell carcinoma of the esophagus. J. Clin. Oncol..

[30] Suazo-Zepeda E., Bokern M., Vinke P.C., Hiltermann T.J.N., De Bock G.H., Sidorenkov G. (2021). Risk factors for adverse events induced by immune checkpoint inhibitors in patients with non-small-cell lung cancer: A systematic review and meta-analysis. Cancer Immunol. Immunother..

[31] Strzelak A., Ratajczak A., Adamiec A., Feleszko W. (2018). Tobacco smoke induces and alters immune responses in the lung triggering inflammation, allergy, asthma and other lung diseases: A mechanistic review. Int. J. Environ. Res..

[32] Hogg J.C. (2006). Why does airway inflammation persist after the smoking stops?. Thorax.

[33] Kim J.W., Kim T.H., Kim J.-H., Lee I.J. (2018). Predictors of post-treatment stenosis in cervical esophageal cancer undergoing high-dose radiotherapy. World J. Gastroenterol..

[34] Werner-Wasik M. (2005). Treatment-related esophagitis. Semin. Oncol..

[35] Vanagunas A., Jacob P., Olinger E. (1990). Radiation-induced esophageal injury: A spectrum from esophagitis to cancer. Am. J. Gastroenterol..

[36] Seaman W.B., Ackerman L.V. (1957). The effect of radiation on the esophagus. Radiology.

[37] Berthrong M., Fajardo L.F. (1981). Radiation Injury in Surgical Pathology. Am. J. Surg. Pathol..

[38] Papazian A., Capron J.P., Ducroix J.-P., Dupas J.-L., Quenum C., Besson P. (1983). Mucosal bridges of the upper esophagus after radiotherapy for Hodgkin’s disease. Gastroenterology.

[39] Handschel J., Sunderkötter C., Prott F.-J., Meyer U., Kruse-Lösler B., Joos U. (2001). Increase of Rm3/1-positive macrophages in radiation-induced oral mucositis. J. Pathol..

[40] Sonis S.T., Peterson R.L., Edwards L.J., Lucey C.A., Wang L., Mason L., Login G., Ymamkawa M., Moses G., Bouchard P. (2000). Defining mechanisms of action of interleukin-11 on the progression of radiation-induced oral mucositis in hamsters. Oral. Oncol..

[41] Deng H.-Y., Alai G., Luo J., Li G., Zhuo Z.-G., Lin Y.-D. (2018). Cancerous esophageal stenosis before treatment was significantly correlated to poor prognosis of patients with esophageal cancer: A meta-analysis. J. Thorac. Dis..

[42] Mariette C., Balon J.M., Maunoury V., Taillier G., Van Seuningen I., Triboulet J.P. (2003). Value of endoscopic ultrasonography as a predictor of long-term survival in oesophageal carcinoma. Br. J. Surg..

[43] Cho C.J., Song H.J., Lee G.H., Choi K.D., Kim Y.-H., Ryu J.S., Kim S.B., Kim J.H., Park S.I., Jung H.Y. (2017). Clinical implications of endoscopic ultrasonography non-traversability in patients with locoregional esophageal cancer receiving multimodality therapy. Korean J. Intern. Med..

[44] Yang Y.S., Hu W.P., Ni P.Z., Wang W.P., Yuan Y., Chen L.Q. (2017). Esophageal luminal stenosis is an independent prognostic factor in esophageal squamous cell carcinoma. Oncotarget.

[45] Rieu D., Filleron M.C., Beluchon T.B. (2013). Recurrence risk after Ivor Lewis oesophagectomy for cancer. J. Cardiothorac. Surg..

