# Supplementary material for: Efficacy and Safety of Immune Checkpoint Inhibitor Combination Therapy for Dysphagia in Patients with Advanced Esophageal Cancer
Source: J Clin Med. 2024 Aug 15;13(16):4806. doi: 10.3390/jcm13164806 (PMC11355245; doi:10.3390/jcm13164806)
Supplement: Supplementary file 1 [file jcm-13-04806-s001.zip › jcm-3150866-supplementary.pdf]

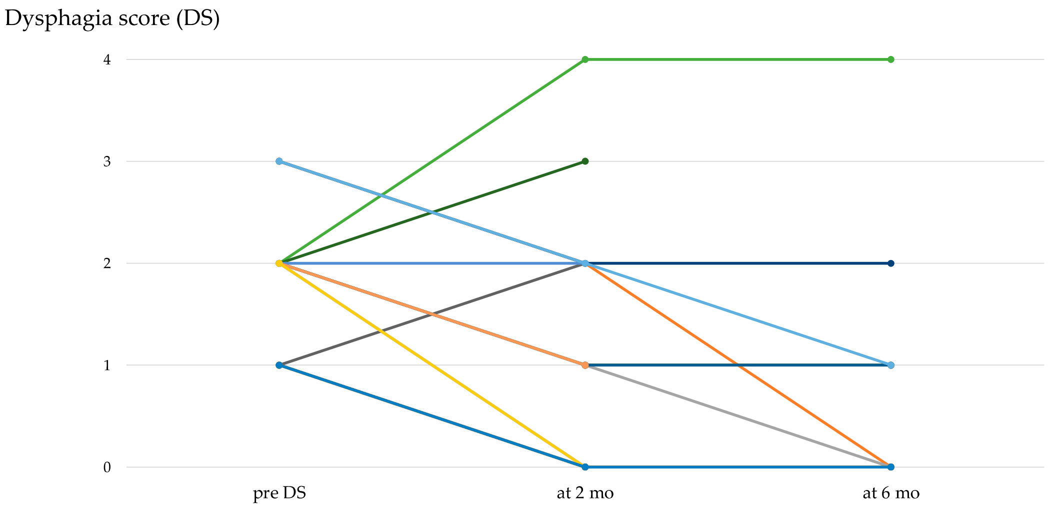

Figure S1. Time course of dysphagia scores during treatment for each cases.
at 2 mo: at 2 months, at 6 mo: at 6 months

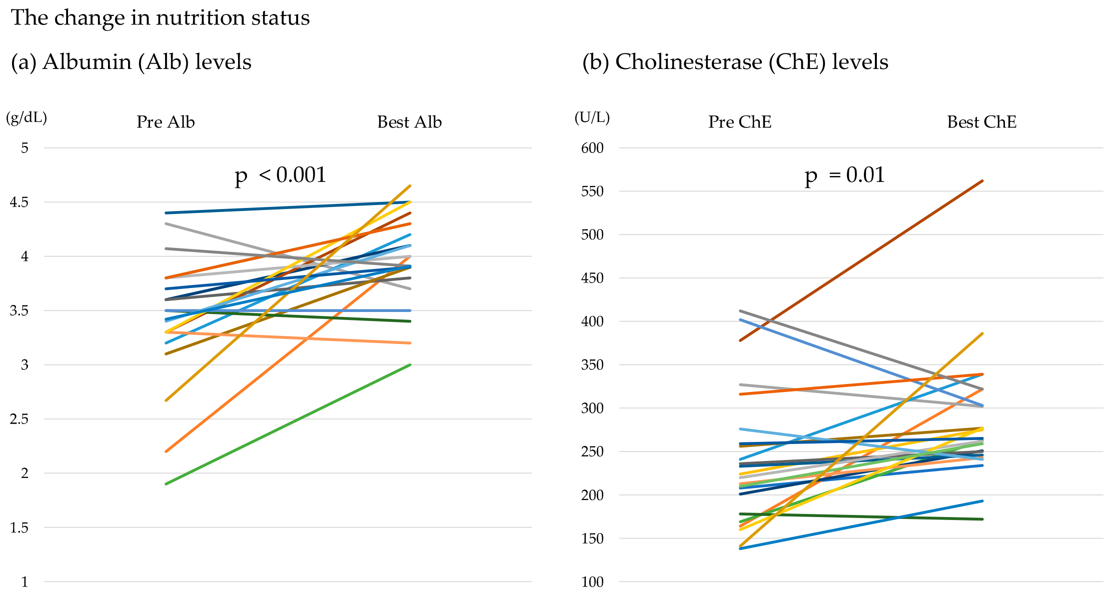

Figure S2. The change in nutrition status (a) the change in albumin levels, (b) the change in cholinesterase levels for each case.
